# Oriental management strategies for urban resilience: Based on the Wuli-Shili-Renli methodology and coupled coordination degree model

**DOI:** 10.1016/j.heliyon.2023.e16279

**Published:** 2023-05-18

**Authors:** Wei Luo, Zhihua Huang, Suiying Cheng, Zhuoqun Gan

**Affiliations:** a Business School, Henan University, Kaifeng, 475000, Henan, China; b Cultural Industry and Tourism Management School, Henan University, Kaifeng, 475000, Henan, China; c Mathematics and Statistic School, Henan University, Kaifeng, 475000, Henan, China

**Keywords:** Urban resilience, Coupled coordination degree model, The Wuli-Shili-Renli methodology, Complex system

## Abstract

Extreme weather is frequent and aggressive, posing a huge challenge to urban management capacity. The construction of urban resilience is a systematic project of multi-system coordination. Previous studies have focused on temporal evolution, external system coupling and coordination, and less on the internal study of urban resilience systems. Based on the perspective of the Wuli-Shili-Renli methodology, the study combines urban resilience with Eastern management philosophy. Using a coupled coordination model, the evolutionary laws of key elements of multiple processes in the complex urban resilience system of Henan Province are studied. The coupled coordination mechanism of multiple elements and processes in the province is revealed. It is found that (1) the development of the urban resilient system in Henan Province has gone through two stages from fluctuation to stability. From 2010 to 2015 called “fluctuating growth” and from 2016 to 2019 called “linear growth”. (2) There are three different periods of development for the coordination of the urban resilient system in Henan. Stage 1 (2010–2015) the “coupling teething period”, stage 2 (2016–2017) the “decoupling accumulation period” and stage 3 (2018–2019) the “self-organized explosive period”. (3) Henan has strong preventive power, but weak resistance and recovery power. Then, from the perspective of WSR, the optimal regulation of the regional urban resilient system is proposed.

## 1 Introduction

Resilience, since first introduced into ecological governance [1], has exploded in many academic fields, such as engineering, business management, agricultural science [2]. Urban resilience as a capacity to help cities cope with external shock events has gained much attention in recent years. Especially since the world has been hit by natural disasters frequently, the governments are proposing to enhance the capacity of urban resilience to cope with it [3].

Urban resilience is an ability to help cities absorb external influences and maintain operations in the event of natural disasters or external shocks [2,[4], [5], [6]]. Different perspectives have various measurements of urban resilience [5,6]. The following dimensions are recognized as important measurement elements by the academic community, including physical, natural, economic, institutional, and social [4]. Baseline Resilience Indicators for Communities (BRIC) theoretical framework was design by Cutter [7] to measure urban resilience, including six dimensions of social, economic, housing and infrastructure, institutional, community, and environmental. The BRIC framework can be applied to assess urban resilience in countries [8], provinces [9,10] and regions [11,12]. The BRIC is universal enough, it can also be used to analyze China's urban resilience. With the rapid urbanization process in China, the increase in population density poses a complex challenge to urban management and sustainability. The significance of collaborative governance of urban resilience from the perspective of complexity.

The Wuli-Shili-Renli methodology was designed to analyze the complex problem from an oriental perspective by Gu and Zhu [13]. It provides a way to divide complex problems into three dimensions: Wuli (Objective existence), Shili (Interaction), and Renli (Subjective motivation) [[14], [15], [16]]. Wuli, which means objectively existing objects, answers the major question of what material constitutes the objective world and maintains the operation of the complex system. Shili, which means the interaction between things, answers the major question of how the system goes. Renli, which means the subjective objects, answers the major question of what we could do to sustain the system running [13,14,16,17]. The WSR methodology has been widely used in many research areas including risk management [18,19], energy management [[20], [21], [22]], public management [23,24], construction management [25,26], tourism management [27], and knowledge management [28]. Wul-Shili-Renli methodology also applies to urban resilience.

This study combines the WSR methodology with the BRIC framework to analyze urban resilience system. And assessed the coupling coordination of urban resilience subsystems in Henan Province. Then proposed an urban resilience enhancement strategy suitable for the management context in China.

## 2 Models and methods

### 2.1 The modified BRIC model

Chen [29] proposed a modified model based on BRIC with the practical experience of Chinese cases. Table 1 showed the dimensions, sub-dimensions, and indicators of a modified BRIC model. The selection of indicators takes into account both theoretical compliance and data accessibility.

**Table 1:** The modified BRIC framework.

Dimensions	Sub-dimensions	Indicator	Effect	Weight
Environmental resilience	Ecosystem	Annual precipitation	Positive	0.124
	Hazard intensity	Direct economic losses from natural disasters	Negative	0.085
	Hazard severity	Greening coverage of built-up areas	Positive	0.227
	Risk and exposure	Urban land construction utilization rate	Positive	0.151
	Sustainability	Sewage treatment rate	Positive	0.189
	Protective resources	Park green space per capita	Positive	0.224
Infrastructure Resilience	Housing type and codes	Building land area per capita	Positive	0.171
	Response and recovery	Beds per capita per 10,000	Positive	0.160
	Transportation network	Road area per capita	Positive	0.182
	Critical infrastructure	Daily domestic water consumption per capita	Positive	0.286
	Infrastructure exposure	Urban water supply capacity	Positive	0.201
Economic Resilience	Economic diversity	The proportion of GDP accounted for by secondary and tertiary industries	Positive	0.160
	Employment	Unemployment rate	Negative	0.154
	Asset	Savings per capita	Positive	0.191
	Income and equality	Disposable income per capita	Positive	0.154
	Economic recovery	Tax revenue as a percentage of fiscal revenue	Positive	0.193
	Resource level	Fiscal revenue	Positive	0.148
Institutional Resilience	Hazard mitigation and plans	Population density	Negative	0.128
	Preparedness	Number of social service vocational skills per 10,000 people	Positive	0.177
	Management, leadership and policy	Public services as a percentage of fiscal expenditure	Positive	0.219
	Managerial resources	Number of beds in social service institutions per 10,000 people	Positive	0.233
	Institutional characters	Number of health facilities per 10,000 people	Positive	0.243
Social resilience	Education and equity	The proportion of illiterate and semi-literate people to the population aged 15 and over	Negative	0.098
	Demography characteristics	Civilian car ownership	Positive	0.210
	Public health services	Practicing (assistant) physicians per 10,000 people	Positive	0.237
	Social services and well-being	Health institutions per 10,000 people	Positive	0.355
	Social security	Public safety as a percentage of fiscal spending	Negative	0.100
Community Capital	Social capital	Number of social workers per 10,000 people	Positive	0.135
	Community competence	Number of neighborhood councils per 10,000 people	Positive	0.111
	Innovation	Share of Scientific Researchers	Positive	0.075
	Community bonds	Mobile population share	Positive	0.210
	Social engagement	Community voting participation rate	Positive	0.229
	Knowledge, information and awareness	Number of teachers per 10,000 people	Positive	0.240

### 2.2 The urban resilience of the WSR approach

**Fig. 1:**
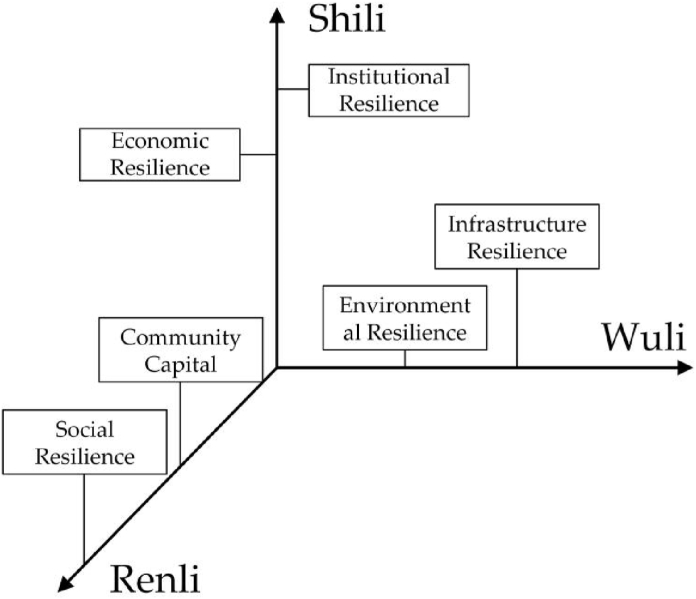
The urban resilience of the WSR approach.

**Fig. 2:**
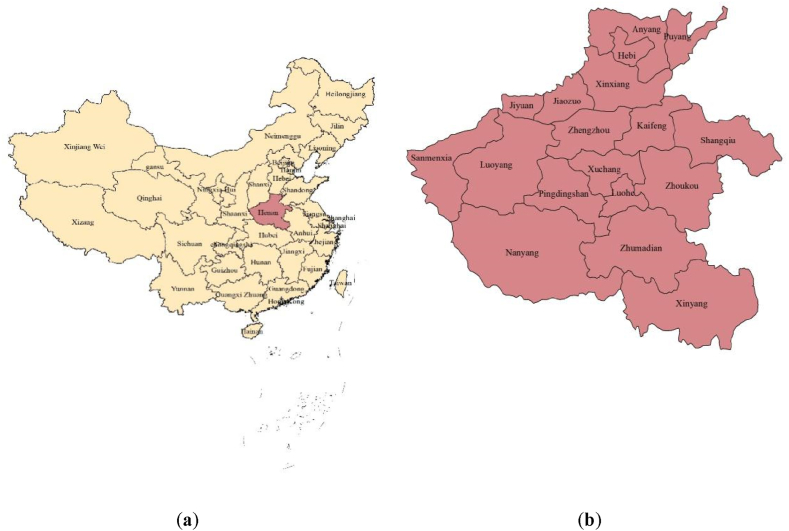
Study area.

Based on the WSR method, the framework of urban resilience analysis is built (Fig. 1). Wuli includes environmental resilience and infrastructure resilience because the ecological environment and infrastructure construction of the city are inherent properties, which are difficult to change in the short term due to management policies or external changes. Shili includes economic resilience and institutional resilience, compared with the Wuli, this dimension is concerned more with the “human” factor, reflecting the combination of emotion and reason, and the city economy and system are affected by the manager's governance ability. Renli, compared with the Shili and Wuli, the human-rational dimension is more emotional, and its attribute changes are closely related to the policy environment. When hit by a disaster, managers can change their attributes through a variety of management tools such as policy formulation and resource deployment to cope with the disaster (see Fig. 2).

### 2.3 The TOPSIS method

TOPSIS method was used for data processing, even there are many ways to define the weights of indicators, the TOPSIS method is the best way to get weights more objectively. It helps integrate the multi-metric quantities of each Sub-dimensions into each dimension. It calculates objective weights by using the information on data characteristics [30]. The entropy represents how chaotic the data is in the set, and it helps define the weights of indicators. The calculation process is as follows [31]:

The collected data are first normalized, and for positive indicators $x_{i}$:

(1) $$y_{i}=\frac{x_{i}-x_{i}^{\min}}{x_{i}^{\max}-x_{i}^{\min}}$$

for negative indicators $x_{i}$:

(2) $$y_{i}=\frac{x_{i}^{\max}-x_{i}}{x_{i}^{\max}-x_{i}^{\min}}$$

where y means the normalized value, i means the indicator serial number. After normalization, the minimum data is defined as 0, which will interfere with the calculation of the logarithm of information entropy. So we used data panning to eliminate the effect, then calculate the information entropy $e_{i}$:

(3) $$z_{i}=\frac{y_{i}}{\sum_{t}y_{i}}$$

(4) $$e_{i}=-\frac{1}{\ln\;t}\sum_{t}\left(z_{i}\;\ln\;z_{i}\right)$$

where z is intermediate quantity, t means times, here t is taken as 10. Then calculated the indicator weights $\omega_{i}$:

(5) $$\omega_{i}=\frac{e_{i}}{\sum_{i^{*}}e_{i}}$$

urban resilience assessment value

(6) $$w_{i^{*}}=\sum_{i^{*}}\omega_{i}\times y_{i}$$

(7) $$W=\left(\sum w_{i^{*}}\right)/6$$

after the progress (1) to (7), the weights of each sub-dimension, the assessment value of each dimension, and the assessment value of urban resilience were got.

### 2.4 The coupling coordination degree model

The coupling coordination degree model is usually used to analyze the coupling and coordination relationship between two systems, and also can be used to analyze multi-composite system problems [32]. Analyze the trend and development path of system operation within the composite system through the calculation of coupling degree and coordination degree. The calculation process is as follows [33,34]:

The coupling degree(*C*) between the six subsystems of urban resilience:

(8) $$C={\left[{\prod w_{i}/\left(\frac{\sum w_{i}}{n}\right)}^{n}\right]}^{\frac{1}{n}}$$

where n is 6. The coordination degree(*D*) between the six subsystems of urban resilience:

(9) $$D=\sqrt{C\times W}$$

Referring to the existing standards and combining with the data characteristics, the coupling coordination level is divided as shown in Table 2.

**Table 2:** The coupling coordination level.

*C*	Coupling Level	*D*	Coordination Level
0.00–0.40	Low-level coupling	0.00–0.20	High-level disorders
		0.21–0.40	Medium-level disorders
0.41–0.60	Medium-level coupling	0.41–0.50	Low-level disorders
		0.51–0.60	Low-level coordination
0.61–0.80	Growth coupling	0.61–0.80	Medium-level coordination
0.81–1.00	High-level coupling	0.81–1.00	High-level coordination

### 2.5 Study area

This study takes Henan Province as the study area. The Yellow River Basin has long met the problems with a fragile ecological landscape, vastly different levels of development, and loose cooperative relationships [35]. Henan Province is located in central China, in the middle reaches of the Yellow River Basin, had suffered a lot because of natural disasters [36]. The 7/20 rainstorm event has shown the weak urban resilience capacity in Zhengzhou, the capital city of Henan Province. As an important area in the Yellow River Basin, it is vital to cultivate disaster mitigation capacity in Henan Province.

### 2.6 Data source

The data were selected from 2010 to 2019 for the development of cities in Henan Province. Due to the lack of data, it is difficult to ensure the reliability of data before 2010. In order to make the research conclusions more extensive, a long research interval is selected as far as possible, so the starting point of the research interval is 2010. And avoid the influence brought by COVID-19, the study interval is before 2020. Data sources are from the EPS data platform. It includes China City Database, China Regional Economic Database, and China Urban and Rural Construction Database. There are also the Henan Statistical Yearbook and the statistical yearbooks of various cities.

## 3 Results

### 3.1 Temporal evolution of urban resilience in Henan Province

Through data acquisition and entropy weighting method, the assessed values of urban resilience and the assessed values of each sub-dimension in Henan Province from 2010 to 2019 are obtained (Table 3).

**Table 3:** Assessed values of urban resilience and each sub-dimension.

Year	Environmental Resilience	Infrastructure Resilience	Economic Resilience	Institutional Resilience	Social Resilience	Community Capital	Urban Resilience
2010	0.149	0.047	0.198	0.769	0.403	0.327	0.316
2011	0.232	0.113	0.267	0.742	0.512	0.482	0.391
2012	0.163	0.120	0.352	0.503	0.272	0.255	0.278
2013	0.244	0.186	0.453	0.578	0.370	0.353	0.364
2014	0.421	0.270	0.473	0.500	0.454	0.259	0.396
2015	0.472	0.388	0.496	0.178	0.421	0.250	0.367
2016	0.619	0.533	0.510	0.088	0.457	0.156	0.394
2017	0.755	0.714	0.654	0.090	0.460	0.321	0.499
2018	0.839	0.849	0.748	0.095	0.540	0.437	0.584
2019	0.868	0.992	0.780	0.335	0.595	0.650	0.703

**Fig. 3:**
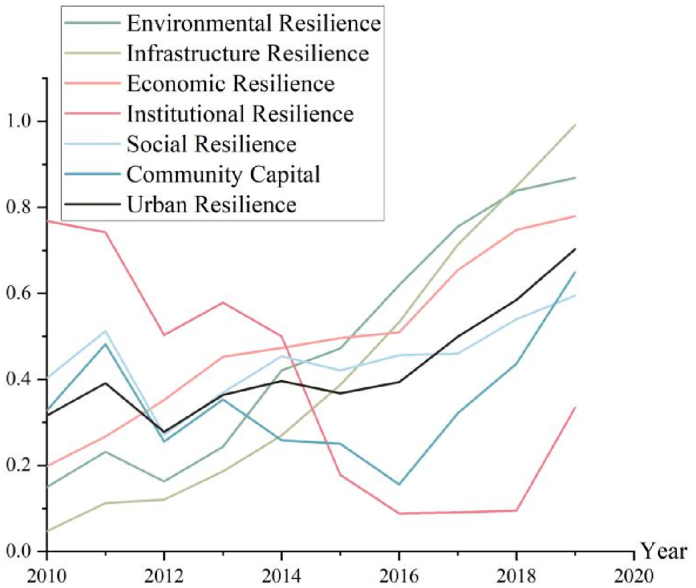
Urban resilience system evaluation value.

In terms of the overall growth path of the system, the resilience of Henan cities has increased significantly over the past decade and has shown a “two-step” trend, with “fluctuating growth” from 2010 to 2015 and “linear growth” from 2016 to 2019. From 2010 to 2015, it showed “fluctuating growth”, and from 2016 to 2019, it showed “linear growth”. From the subsystem development curve, environmental resilience, facility resilience, and economic resilience synergize more obviously and remain similar to the urban resilience development curve, while social resilience and community capital grow more slowly, coupled with the imbalance of institutional resilience development and other constraints, to a certain extent inhibit the overall growth rate of urban resilience (see Fig. 3).

### 3.2 Coupling coordination of urban resilience in Henan Province

**Fig. 4:**
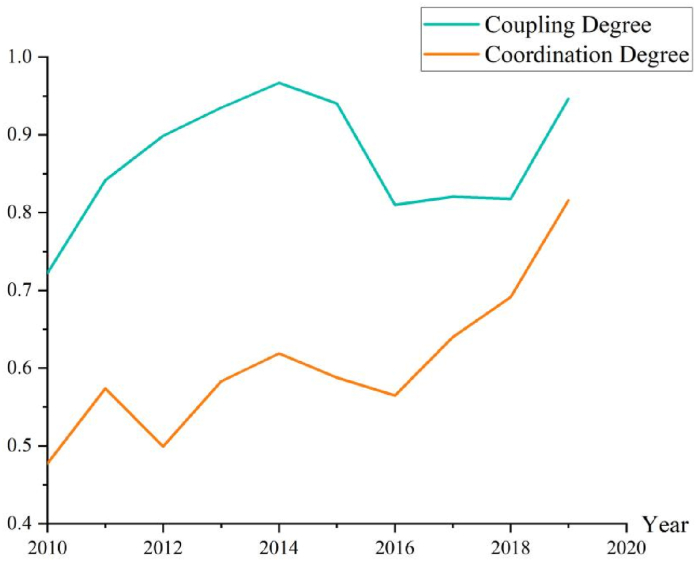
The coupling coordination temporal evolution.

Through the coupling coordination degree model, the coupling coordination degree and grade evaluation of the urban resilience system of Henan during 2010–2019 were calculated (Table 4). Fig. 4 showed the temporal trend of the coupling degree (histogram) and the coordination degree (line).

**Table 4:** The coupling coordination degree and level.

Year	Coupling Degree	Coordination Degree	Coupling Level	Coordination Level
2010	0.722	0.477	Growth coupling	Low-level disorders
2011	0.842	0.574	High-level coupling	Low-level disorders
2012	0.899	0.499	High-level coupling	Low-level disorders
2013	0.935	0.583	High-level coupling	Low-level disorders
2014	0.967	0.619	High-level coupling	Low-level coordination
2015	0.940	0.588	High-level coupling	Low-level coordination
2016	0.810	0.565	High-level coupling	Low-level coordination
2017	0.820	0.640	High-level coupling	Medium-level coordination
2018	0.818	0.691	High-level coupling	Medium-level coordination
2019	0.946	0.816	High-level coupling	High-level coordination

It can be divided into three stages. Stage 1 (2010–2015), called “coupling teething period (2010–2015)", the coupling degree was high-level but the coordination degree was low-level. The components of the urban resilience system are being put together and have not yet to form a consistent pace. Stage 2 (2016–2017), called “decoupling accumulation period”, reduced nearly to the growth coupling level, and the coordination degree was increasing to medium-level. System components are reducing coupling and finding opportunities to work together. And Stage 3 (2018–2019), called “self-organized explosion period”, both coupling and coordination degrees were growth to high-level. Each component of the system has found its own optimal development mode, and the whole system is in a highly coordinated state.

## 4 Discussions

From the perspective of time series, it has been confirmed that the evaluation value of urban resilience in Henan Province increases year by year [37]. Next, we analyze the degree of coupling and coordination of each part separately from the WSR perspective. Then further analysis is done from the three different system phases shown in the results.

### 4.1 The WSR approach to urban resilience

Based on the WSR approach, the BRIC framework is divided into three parts, Wuli, Shili, and Renli. The Wuli contains environment resilience and infrastructure resilience. The Shili contains economic resilience and institutional resilience. The Renli contains social resilience and community capital. The analysis unfolded in each part helps to discover the rich coupling in the development process of urban resilience.

#### 4.1.1 The Wuli level growth solidly

**Fig. 5:**
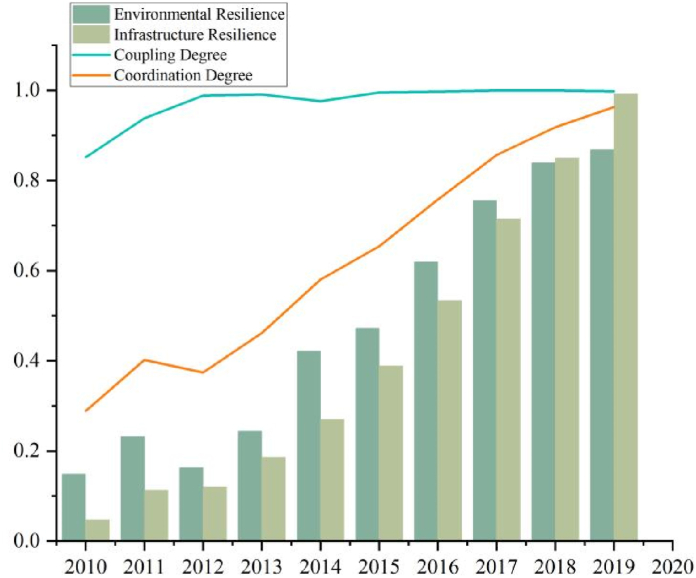
The Wuli level of evaluation value and coupling coordination degree.

The Wuli level (Fig. 5) contains environmental resilience and facility resilience, which are inherent properties of the objective level, and it is difficult to respond quickly in the short term while building a physical barrier to prevent disasters that are the preventive power of the city. The overall growth of environmental resilience and facility resilience is relatively strong, with values increasing from 0.149 to 0.047 to 0.868 and 0.992, respectively, and showing a perfect growth curve. The coupling degree is maintained at a high level and the coordination degree is increasing linearly. Thanks to the rapid economic development, urban management has increased the investment in the construction of infrastructure and other environmental aspects, such as sponge city construction project, comprehensive pipeline corridor construction, and construction of sewage treatment facilities, which has increased the environmental and ecological carrying capacity and laid a solid foundation for the construction of urban resilience in Henan. When disaster strikes, urban green areas, parks, and other public places can be used as emergency evacuation sites to enhance the city's emergency protection capacity, while the per capita road area and water supply capacity guarantee the normal operation of residents' production and life, and the annual precipitation has not yet formed a benign development by natural factors.

#### 4.1.2 The Shili level development imbalance

**Fig. 6:**
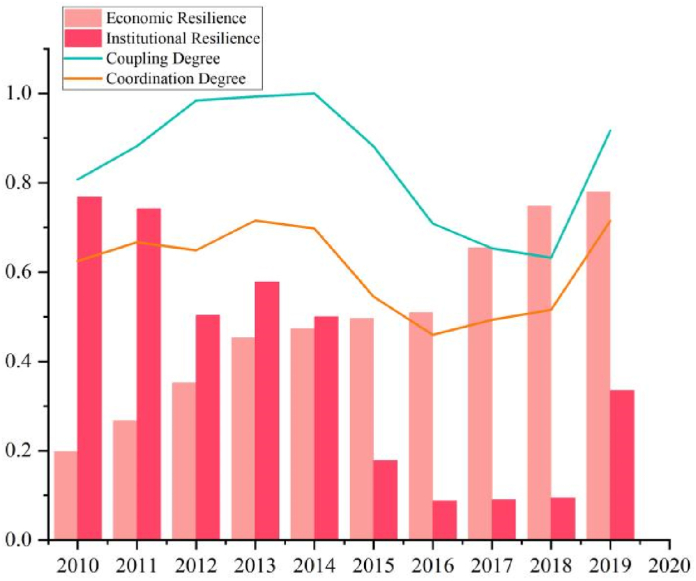
The Shili level of evaluation value and coupling coordination degree.

The Shili level (Fig. 6) contains economic resilience and institutional resilience, which is between the subjective and objective principles of urban operation, in the short term with a certain degree of flexibility, the buffer zone is constructed when a disaster comes that the city's resistance. Economic resilience and institutional resilience growth diverge, Henan's economic development level has increased year by year, making economic resilience grow from 0.198 to 0.780, forming a better growth curve and acting as a power source of urban resilience to inject strong vitality into the growth of urban resilience in Henan. Shili's coupling and coordination degrees kept the same fluctuations as the whole system, with inflection points in 2016 and 2018. Good economic resilience guarantees the normal functioning of society, while a higher level of resilience can provide a superior external environment for economic development. The increase in the proportion of the secondary tertiary industry, disposable income, tax revenue, and fiscal revenue year by year constructs a stronger disaster response buffer. Good institutional resilience reflects the concept of urban management, and social service agencies and personnel, and health institutions construct the life-safety line for the affected groups. Along with the rapid urbanization, the unemployment rate is rising, the population density is increasing, and the investment in public services, such as health institutions and social services, is insufficient.

#### 4.1.3 The Renli level growth slowly

**Fig. 7:**
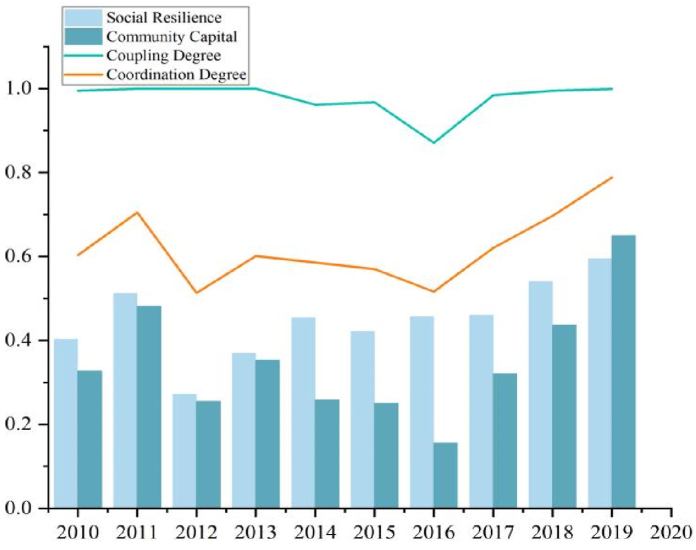
The Renli level of evaluation value and coupling coordination degree.

The Renli level (Fig. 7) contains urban social resilience and community capital, which reflect the subjective level governance concept of urban managers and form the self-healing ability of the city to recover actively, i.e., the resilience of the city. Social resilience and community capital show a flat growth trend, from 0.327 to 0.316 to 0.650 and 0.703 respectively. Their growth rate and evolution space are smaller than the resilience assessment value of the environment, facilities, and economic subsystems, and the construction of social resilience and community capital has a certain foundation in 2010. Renli's coupling degree has remained relatively good, with minor fluctuations in 2016. Its coordination degree was relatively low until 2016 and only improved from 2016. When a disaster strikes, teachers, doctors, researchers, public officials, and other educated groups can assist in disaster mitigation under the unified command of the emergency management center, enhancing the government's rapid execution and enabling the affected area to recover quickly. Although the total population of Henan has not changed much during the decade, the flow of people from rural to urban areas has never stopped, and the increasing density of the urban population, the increase in mobile population, and the decrease in social participation have tested the social carrying capacity of cities.

### 4.2 Three stages of coupling coordination

#### 4.2.1 Stage one “the coupling teething period”

2010–2015 is the “coupling teething period”, in which the coupling degree between subsystems is continuously improved, and the mutual linkage between them is continuously improved and formed into a community, but the system as a whole has not yet formed a good development trend, and the overall coordination degree fluctuates at the critical level of disorder and coordination, and the urban resilience. The assessment value of urban resilience has not increased significantly in this stage. Environmental resilience, infrastructure resilience, and economic resilience have been steadily increasing, while social resilience is at a low to medium level and has not been improved to a large extent, institutional resilience and community capital have deteriorated, and urban management has not yet intervened in “governance of Shili” and “governance of Renli”. The urban resilience construction has only improved the environment and facilities at the objective level but has not yet achieved a qualitative breakthrough.

#### 4.2.2 Stage two “the decoupling accumulation period”

2016–2017 is the “decoupling accumulation period”, the coupling degree between subsystems has decreased to the growth coupling level, the overall coordination of the system has increased and developed into medium-level coordination, and the evaluation value of urban resilience has increased to a certain extent. Environmental resilience, facility resilience, and economic resilience have grown steadily, social resilience and community capital have improved compared with the previous stage, and the level of urban resilience has improved after more “governance of Renli” means have been involved in urban management, achieving a breakthrough from 0 to 1.

#### 4.2.3 Stage three “the self-organization explosion period”

2018–2019 is the “self-organization explosion period”, the coupling degree is high level, the correlation degree among subsystems is highly correlated, the system coordination degree is high-level coordination, and the overall urban resilience system is highly unified and coordinated, forming a positive feedback linkage co-evolutionary consortium, and the city. The city resilience evaluation value increases the most. From the subsystem level, the evaluation value of each subsystem has increased substantially, and the combination of “physical governance”, “human governance” and “rational governance” has achieved a qualitative breakthrough of “three-tier The combination of “governance of Wuli”, “governance of Shili” and “governance of Renli” has achieved a qualitative breakthrough of “unity".

In summary, the development of the Henan urban resilience system is in line with the evolution trend of complex systems, and has gone through three processes of coupling-decoupling-coupling, achieving good coordination among systems, and has entered the process of self-organization of urban resilience complex systems. The development pattern of urban resilience system at the level of Henan Province and the Yellow River Basin is similar [38]. In the future, when the high-intensity coupling reaches the bottleneck of system development, we should take the initiative to intervene to take decoupling means, find the weak point of urban resilience construction, and realize the dynamic coordination of systems again.

### 4.3 Limitations

This study analyzes the urban resilience development from the perspective of complex systems. In the future, the inter-regional network effects within the basin should be considered, and a more detailed path analysis of inter-provincial and inter-watershed urban resilience development should be formed by combining various analysis methods such as QCA. And due to the influence of COVID-19 and lack of data, the research range was limited to 10 years, and the generalization of the research conclusions was insufficient.

## 5 Conclusions and policy implications

### 5.1 Conclusions

Based on the BRIC model, we measured the evaluation value, coupling degree, and coordination degree of the urban resilience system in Henan Province in the past ten years, analyzed the development of the urban resilience complex system using the WSR method, and obtained the following conclusions.

(1) Over the past decade, the spatial and temporal evolution of urban resilience in Henan has improved significantly. In terms of the overall growth path of the system, it shows a “fluctuating growth” (2010–2015) followed by a “linear growth” (2016–2019). “From the subsystem development curve, the synergy of environmental resilience, infrastructure resilience, and economic resilience is obvious and remains similar to the urban resilience development curve, while the growth of social resilience and community capital is slower, and there is still some imbalance in the development of institutional resilience.
(2) Over the past decade, the coupling and coordination of the urban resilience system in Henan has undergone a “coupling teething period” (2010–2015), a “decoupling accumulation period” (2016–2017), and a “self-organizational explosion period”.), and the “self-organized explosive period” (2018–2019), and entered the self-organized positive-order synergy state.
(3) Based on the WSR perspective, the Wuli constructs urban preventive force, the Shili constructs urban resistance, and the Renli constructs urban resilience. Henan's urban resilience system as a whole has strong preventive power, moderate resistance, and moderate recovery power.

### 5.2 Policy implications

The above analysis and conclusions lead to the following policy recommendations.

(1) Maintain the advantage of “governance of Wuli”. By strengthening environmental construction, improving the system of guaranteeing water resources allocation in the whole area of the Yellow River, and dealing with the relationship between industrial water, agricultural water, and domestic water to avoid exchanging ecology for development [39]. At the same time, maintain the advantage of infrastructure construction, promoting green buildings [40,41], consolidate the leading level of highway access in the province, accelerate the construction of public infrastructure in the new era such as 5G, big data, and the Internet of things, improve the level of scientific and technological governance, and strengthen the “preventive power” of the city.
(2) Improve the level of “governance of Shili”. By maintaining the level of economic resilience construction, giving full play to the location advantage, expanding the degree of openness, seizing the opportunity of international logistics airport construction, corresponding to the Central Plains city cluster and the Yellow River development strategy, and enhancing the economic primacy of the basin. At the same time, enhance the construction of the urban resilience system, and build the municipal, district, and street-level social service institutions with comprehensive coverage. Improve the level of medical security and social security, and improve the social security system, so that it can keep up with the population density. Maintain year-on-year growth. The redundancy of social service institutions will be improved to enhance urban resilience.
(3) Strengthen the “governance of Renli”. Expand the investment in science, education, culture, and health, build several vocational training schools from primary, and intermediate to advanced levels, improve the level of higher education, improve the regional education level, reduce the illiteracy rate, and create more jobs for people with higher education. At the same time, intensify the introduction of talents, attract talents to settle in the city, cultivate the mentality of a “master”, improve the level of social participation, and strengthen the social identity. Improve the level of participation in social governance, and improve the “governance of Renli” [42]. In turn, the government's responsiveness will be improved and the urban resilience will be enhanced.
(4) Improve the management level of urban resilience and balance the decoupling and coupling process. From the perspective of complex system theory, Henan urban resilience system has entered the stage of good level development, and the high coupling and high coordination among the subsystems will promote the positive evolution of the resilience system in the coming period. But should avoid the trap of “governance by doing nothing”, we still need to pay close attention to the development of urban resilience subsystems, and improve the level of the emergency management system [43]. Intervene in times when the system is out of balance. Balance the process of “governance by doing nothing” and “active intervention”, and systematically build up a strong preventive, resilient, and restorative power of the city.

## Author contribution statement

Wei Luo & Suiying Cheng: Conceived and designed the experiments.

Zhihua Huang: Analyzed and interpreted the data; Contributed reagents, materials, analysis tools or data; Wrote the paper.

Zhuoqun Gan: Performed the experiments.

## Data availability statement

Data will be made available on request.

## Additional information

No additional information is available for this paper.

## Credit authors statement

**Wei Luo:** Conceptualization, Methodology, Writing - review & editing, Investigation. **Zhihua Huang:** Formal analysis, Writing - original draft. **Suiying Cheng:** Investigation, Funding acquisition, Writing – review & editing. **Zhuoqun Gan:** Data curation.

## Funding

This research was supported by the Chinese National Funding of Social Sciences [grant number 21ZDA081]; 10.13039/100018979Henan Office of Philosophy and Social Science [grant number 2022BJJ033]; Henan Province key R & D and promotion special project (soft science) [grant number 222400410140]; Henan Province key R & D and promotion special project (soft science) [grant number 222400410635]; Henan Province education science “14th Five-year plan” 2021 general project [grant number 2021YB0034].

## Declaration of competing interest

The authors declare that they have no known competing financial interests or personal relationships that could have appeared to influence the work reported in this paper.

## References

[1] Holling C.S. (1973). Resilience and stability of ecological systems. Annu. Rev. Ecol. Systemat..

[2] Meerow S., Newell J.P., Stults M. (2016). Defining urban resilience: a review. Landsc. Urban Plann..

[3] Spaans M., Waterhout B. (2017). Building up resilience in cities worldwide - rotterdam as participant in the 100 resilient cities programme. Cities.

[4] Gomes Ribeiro P.J., Pena Jardim Goncalves L.A. (2019). Urban resilience: a conceptual framework. Sustain. Cities Soc..

[5] Zhao R., Fang C., Liu H. (2020). Progress and prospect of urban resilience research. Prog. Geogr..

[6] Yang X., Wang L., Li Y., Hou Y., Niu J. (2021). Review and prospects of resilient city theory. Geogr. Geo-Inf. Sci..

[7] Cutter S.L., Ash K.D., Emrich C.T. (2014). The geographies of community disaster resilience. Global Environmental Change-Human and Policy Dimensions.

[8] Scherzer S., Lujala P., Rod J.K. (2019). A community resilience index for Norway: an adaptation of the Baseline Resilience Indicators for Communities (BRIC). Int. J. Disaster Risk Reduc..

[9] Javadpoor M., Sharifi A., Roosta M. (2021). An adaptation of the Baseline Resilience Indicators for Communities (BRIC) for assessing resilience of Iranian provinces. Int. J. Disaster Risk Reduc..

[10] Sung C.H., Liaw S.C. (2020). A GIS approach to analyzing the spatial pattern of baseline resilience indicators for community (BRIC). Water.

[11] Singh-Peterson L., Salmon P., Goode N., Gallina J. (2014). Translation and evaluation of the baseline resilience indicators for Communities on the sunshine coast, queensland Australia. Int. J. Disaster Risk Reduc..

[12] Nafishoh Q., Riqqi A., Meilano I. (2016).

[13] Gu J.F., Zhu Z.C. (2000). Knowing wuli, sensing shili, caring for renli: methodology of the WSR approach. Syst. Pract. Action Res..

[14] Midgley G., Gu J.F., Campbell D. (2000). Dealing with human relations in Chinese systems practice. Syst. Pract. Action Res..

[15] Zhu Z.C. (2000). Dealing with a differentiated whole: the philosophy of the WSR approach. Syst. Pract. Action Res..

[16] Brugha C.W. (2001). Systemic thinking in China: a meta-decision-making bridge to Western concepts. Syst. Pract. Action Res..

[17] Zhu Z.C. (2000). WSR: a systems approach for information systems development. Syst. Res. Behav. Sci..

[18] Wu Y., Chen Z., Wang Z., Chen S., Ge D., Chen C., Jia J., Li Y., Jin M., Zhou T., Wang F., Hu L. (2019). Nuclear safety in the unexpected second nuclear era. Proc. Natl. Acad. Sci. U.S.A..

[19] Luo M. (2021). Establishment of social stability risks evaluation model based on GAHP and IVHFS. J. Intell. Fuzzy Syst..

[20] Ji B., Liu Y., Jin Z. (2018). An evaluation of the design and construction of energy management platform for public buildings based on WSR system approach. Kybernetes.

[21] Li G., Liu J.-G., Wang X.-M., Liu R.-F. (2017). Analysis of influencing factors of change of manufacturing energy intensity in China based on WSR system methodology and VAR model. Eurasia J. Math. Sci. Technol. Educ..

[22] Wang Q., Li S. (2019). Shale gas industry sustainability assessment based on WSR methodology and fuzzy matter-element extension model: the case study of China. J. Clean. Prod..

[23] Chen J., Wang D. (2021). Government credit risk assessment of non-profit public-private partnership projects in China based on the IVHFSs-IFAHP model. Sci. Iran..

[24] Gu J., Xu S., Fang Y., Shi K., Lv B., Peng G., Wang B., Song L., Xie R. (2013). Three aspects on solving queuing service system in Shanghai world expo. J. Syst. Sci. Syst. Eng..

[25] Ye M., Wang J., Si X., Zhao S., Huang Q. (2022). Analysis on dynamic evolution of the cost risk of prefabricated building based on DBN. Sustainability.

[26] Wan P., Wang J., Liu Y., Lu Q., Yuan C. (2022). On risk probability of prefabricated building hoisting construction based on multiple correlations. Sustainability.

[27] Zuo Y., Chen H., Pan J., Si Y., Law R., Zhang M. (2021). Spatial distribution pattern and influencing factors of sports tourism resources in China. ISPRS Int. J. Geo-Inf..

[28] Lin X., Zhang Q., Han X. (2009). Application of Wuli-Shili-Renli system methodology in knowledge management. Kybernetes.

[29] Chen Y., Huang Y., Li K., Luna-Reyes L. (2019).

[30] Omrani H., Alizadeh A., Emrouznejad A. (2018). Finding the optimal combination of power plants alternatives: a multi response Taguchi-neural network using TOPSIS and fuzzy best-worst method. J. Clean. Prod..

[31] Gao Y.P., Chen W.J. (2021). Study on the coupling relationship between urban resilience and urbanization quality-A case study of 14 cities of Liaoning Province in China. PLoS One.

[32] Wei W., Liu X., Wang X., Zhang H. (2021). Spatial heterogeneity of the coupling coordination degree of beautiful China system. Econ. Geogr..

[33] Li G., Zhou Y., Liu F., Wang T. (2021). Regional differences of manufacturing green development efficiency considering undesirable outputs in the yangtze river economic belt based on super-SBM and WSR system methodology. Front. Environ. Sci..

[34] Geng Y.Q., Wang R., Wei Z.J., Zhai Q.H. (2021). Temporal-spatial measurement and prediction between air environment and inbound tourism: case of China. J. Clean. Prod..

[35] Ren B. (2020). The particularity and mode selection of high quality development of the Yellow River Basin. J. Humanit..

[36] Yang Y., Mu Y., Zhang W. (2020). Basic conditions and core strategies of high-quality development in the Yellow River Basin. Resour. Sci..

[37] Liu L., Luo Y., Pei J., Wang H., Li J., Li Y. (2021). Temporal and spatial differentiation in urban resilience and its influencing factors in Henan Province. Sustainability.

[38] Luo W., Huang Z.H., Cheng S.Y., Gan Z.Q. (2022). Study on the coordination between urban resilience and economic development level in the Yellow River Basin. Yellow River.

[39] Mou Y., Luo Y.Y., Su Z.R., Wang J., Liu T. (2021). Evaluating the dynamic sustainability and resilience of a hybrid urban system: case of Chengdu, China. J. Clean. Prod..

[40] Fu X., Hopton M.E., Wang X.H. (2021). Assessment of green infrastructure performance through an urban resilience lens. J. Clean. Prod..

[41] Ling T.Y., Chiang Y.C. (2018). Well-being, health and urban coherence-advancing vertical greening approach toward resilience: a design practice consideration. J. Clean. Prod..

[42] Li Y., Kappas M., Li Y.F. (2018). Exploring the coastal urban resilience and transformation of coupled human-environment systems. J. Clean. Prod..

[43] Wang Z., Deng X.Z., Wong C., Li Z.H., Chen J.C. (2018). Learning urban resilience from a social-economic-ecological system perspective: a case study of Beijing from 1978 to 2015. J. Clean. Prod..

